# Targeting the heart-immune axis after myocardial infarction: from inflammation to immunomodulation

**DOI:** 10.3389/fcvm.2026.1827666

**Published:** 2026-05-28

**Authors:** Li Huang, Riping Xu, Junyu Fan, Liangqing Zhang, Min Chen

**Affiliations:** 1 Faculty of Chinese Medicine and State Key Laboratory of Quality Research in Chinese Medicines, Macau University of Science and Technology, Macau, Macau SAR, China; 2 The Department of Anesthesiology, Affiliated Hospital of Guangdong Medical University, Zhanjiang, China; 3 The Department of Coronary Heart Disease, Affiliated Hospital of Guangdong Medical University, Zhanjiang, China; 4 The Second Affiliated Hospital of Guangdong Medical University, Zhanjiang, China

**Keywords:** crosstalk, heart-immune axis, immunomodulation, inflammation, myocardial infarction

## Abstract

Myocardial infarction (MI) triggers a systemic immune response involving bidirectional crosstalk between the injured heart and remote immune organs. However, the dynamic, multi-organ nature of this “heart-immune axis” remains poorly defined. By targeting specific aspects of the immune response, particularly the modulation of macrophage polarization and the attenuation of excessive inflammation, this strategy holds therapeutic potential for preserving cardiac function and enhancing long-term patient outcomes, thereby opening a new frontier in cardiovascular therapeutics. In this review, we propose a novel framework that divides post-MI immune response into three interconnected phases: inflammation, resolution, and remodeling. Within each phase, we delineate the bidirectional crosstalk mechanisms between the injured heart and immune organs via cytokine networks and hematopoietic progenitor mobilization. We then critically evaluate emerging immunomodulatory strategies, ranging from targeting CCR2/CCL2 axis, promoting regulatory T cells, with emphasis on their mechanistic rationale, clinical trial progress, and major translational gaps. Unlike conventional anti-inflammatory approaches that suppress broad immune activity, axis-targeted immunomodulation aims to preserve or restore protective cardiac repair. This review provides a phase-specific, multi-organ, and therapeutically actionable roadmap of the heart-immune axis, offering new perspectives for next-generation MI therapies.

## 1 Introduction

MI remains a leading cause of global mortality worldwide, is characterized by acute myocardial ischemia resulting from coronary artery occlusion. It is primarily caused by the acute occlusion of a coronary artery, which interrupts blood flow to the myocardial tissue, resulting in ischemic necrosis and posing a significant risk of mortality (1). MI arises from long-term interactions between genetic susceptibility and environmental exposures. Globally, the incidence and mortality rates of acute myocardial infarction (AMI) remain persistently high, predominantly affecting middle-aged and elderly populations, as well as patients with underlying cardiac conditions or high-risk factors such as smoking, diabetes mellitus, and hyperlipidemia (Table 1) (6, 7).

**Table 1:** Immune mechanisms and potential therapeutic targets associated with major MI risk factors.

Risk factor	Immune mechanism	Potential target/intervention
Hypertension	Mechanical stress → endothelial activation → VCAM1/ICAM1 ↑ → monocyte adhesion	Angiotensin II blockade (ARB/ACEi), anti-inflammatory cytokines
Hyperlipidemia	OxLDL → foam cell formation, TLR activation, T cell (Th1) response	Statins (pleiotropic), PCSK9 inhibitors (2), anti-IL-1β
Diabetes	AGEs/RAGE → ROS, NLRP3 inflammasome, pro-inflammatory macrophages	SGLT2 inhibitors, metformin (anti-inflammatory) (3), RAGE antagonists
Smoking	Oxidative stress → neutrophil recruitment, NETosis, endothelial dysfunction	Antioxidants, CXCR2 inhibitors (preclinical), smoking cessation
Obesity	Adipose tissue inflammation → M1 macrophages, leptin/ adipokines, systemic low-grade inflammation	Weight loss, GLP-1 agonists (4), CCR2 antagonists
Gender	Estrogen modulates M2 polarization, reduces neutrophil infiltration; testosterone may promote inflammation	Sex-specific immunomodulation (5); further research needed
Genetic factors	Genetic factors Variants in IL6, TNF, NLRP3 pathways; altered immune cell function	Personalized medicine, genetic risk scores; Polygenic risk scores available (6); no specific drug approved solely based on these variants
Exercise (protective)	Promotes M2 macrophage polarization, increases Tregs, reduces systemic inflammation (↓IL-6, ↓TNF-α) Exercise mimetics, β-adrenergic modulation	Exercise mimetics (5, 6): preclinical/early clinical

VCAM1, vascular cell adhesion molecule 1; ICAM1, intercellular adhesion molecule 1; ACE, angiotensin-converting enzyme; ARB, angiotensin II receptor blocker; OxLDL, oxidized low-density lipoprotein; TLR, Toll-like receptor; Th1, T helper 1; AGE, advanced glycation end product; RAGE, receptor for AGE; ROS, reactive oxygen species; NLRP3, NOD-like receptor family pyrin domain containing 3; SGLT2, sodium-glucose cotransporter 2; NETosis, neutrophil extracellular trap formation; GLP-1, glucagon-like peptide-1; CCR2, C-C chemokine receptor type 2; Treg, regulatory T cell.

Beyond its local mechanical insult, MI immediately triggers a systemic immune response. Atherosclerosis, the underlying cause of most MI cases, is itself an inflammatory process driven by plaque-resident macrophages and T cells (8). Plaque rupture or erosion can abruptly trigger platelet aggregation and intracoronary thrombus formation, leading to vessel occlusion. Importantly, the resulting cardiomyocyte necrosis releases abundant damage-associated molecular patterns (DAMPs), which activate innate immune receptors and initiate a coordinated inflammatory cascade that extends far beyond the heart (9, 10).

The primary therapeutic goal in AMI is rapid restoration of coronary blood flow via pharmacological interventions (antiplatelet agents, anticoagulants, lipid-lowering drugs) and revascularization procedures such as percutaneous coronary intervention (PCI) or emergency coronary artery bypass grafting (CABG) (Table 2) (11, 12). While these strategies have significantly reduced acute mortality, reperfusion itself paradoxically induces sterile inflammation known as ischemia-reperfusion (I/R) injury, which activates remote immune organs (e.g., spleen and bone marrow) through chemokine signals and sympathetic pathways, thereby amplifying systemic immune activation and exacerbating myocardial damage (1, 20). This recognition has shifted attention toward understanding how the heart communicates with distant immune compartments after MI.

**Table 2:** Current therapies for MI and their immune links.

Therapy Category	Specific Methods	Immune/Inflammatory Response	Involved cell types	Immunomodulation Implication
Reperfusion	PCI, thrombolysis, emergency CABG (11, 12)	I/R injury: DAMPs, complement, ROS	Neutrophils, monocytes/macrophages, endothelial cells	Anti-inflammatory adjuncts (e.g., complement inhibitors) at reperfusion
Antiplatelet/Anticoagulant	DAPT, GPIIb/IIIa inhibitors, heparin (13, 14)	Platelet activation →thrombo-inflammation	Platelets, neutrophils, monocytes	Combine with low-dose colchicine to lower residual risk
Secondary Prevention	β-blockers, ACEi/ARB, statins, SGLT2i (15–18)	β-blockade ↓ bone marrow output; RAS inhibitors ↓ NF-*κ*B; statins pleiotropic	Monocytes, macrophages, T cells, HSPCs	Already immunomodulatory; add anti-IL-1β in high-risk patients
Device/Surgery	CABG (19), ICD	CABG: surgical trauma →cytokine release (IL-6, TNF-α)	Neutrophils, monocytes (CABG); none for ICD	Perioperative anti-inflammatory protocols for CABG

DAPT, dual antiplatelet therapy; HSPC, hematopoietic stem/progenitor cell; ICD, implantable cardioverter-defibrillator.

Emerging evidence indicates that the post-MI inflammatory cascade involves complex, bidirectional crosstalk between the injured heart and multiple immune organs, including the spleen (21), bone marrow (22), lymph nodes, and thymus. This multilevel inter-organ network is orchestrated by inflammatory cytokines, DAMPs, and neural reflexes (23–25). For instance, MI rapidly mobilizes splenic monocytes to the infarct zone, while sustained sympathetic activation drives myelopoiesis in the bone marrow, contributing to systemic inflammation and adverse remodeling. Thus, the heart-immune axis represents a dynamic, phase-specific regulatory system that evolves from acute inflammation to resolution and finally to fibrotic remodeling.

In this review, we propose a phase-specific framework for the post-MI heart-immune axis, covering three stages: inflammation, resolution, and remodeling. Unlike previous reviews that focus on individual immune cells or single organs, our framework expands the concept to include the spleen, lymph nodes, and thymus, and explicitly addresses bidirectional heart-immune feedback loops. We first summarize the temporal dynamics of neutrophils, monocytes/macrophages, and lymphocytes following MI. Next, we dissect the molecular mechanisms of heart-immune organ crosstalk. Finally, we critically evaluate immunomodulatory strategies targeting this axis, from mechanistic insights (e.g., macrophage polarization, Treg expansion) to clinical progress (e.g., anti-IL-1β, CCR2 antagonists) and key translational challenges (timing, dosage, comorbidities). In the following sections, we detail the temporal dynamics of immune cells, the bidirectional signaling pathways between the heart and remote immune organs, and the translational opportunities and obstacles in immunomodulation, with the goal of guiding future axis-directed cardiac repair strategies.

## 2 Response of immune system in MI

**Figure 1:**
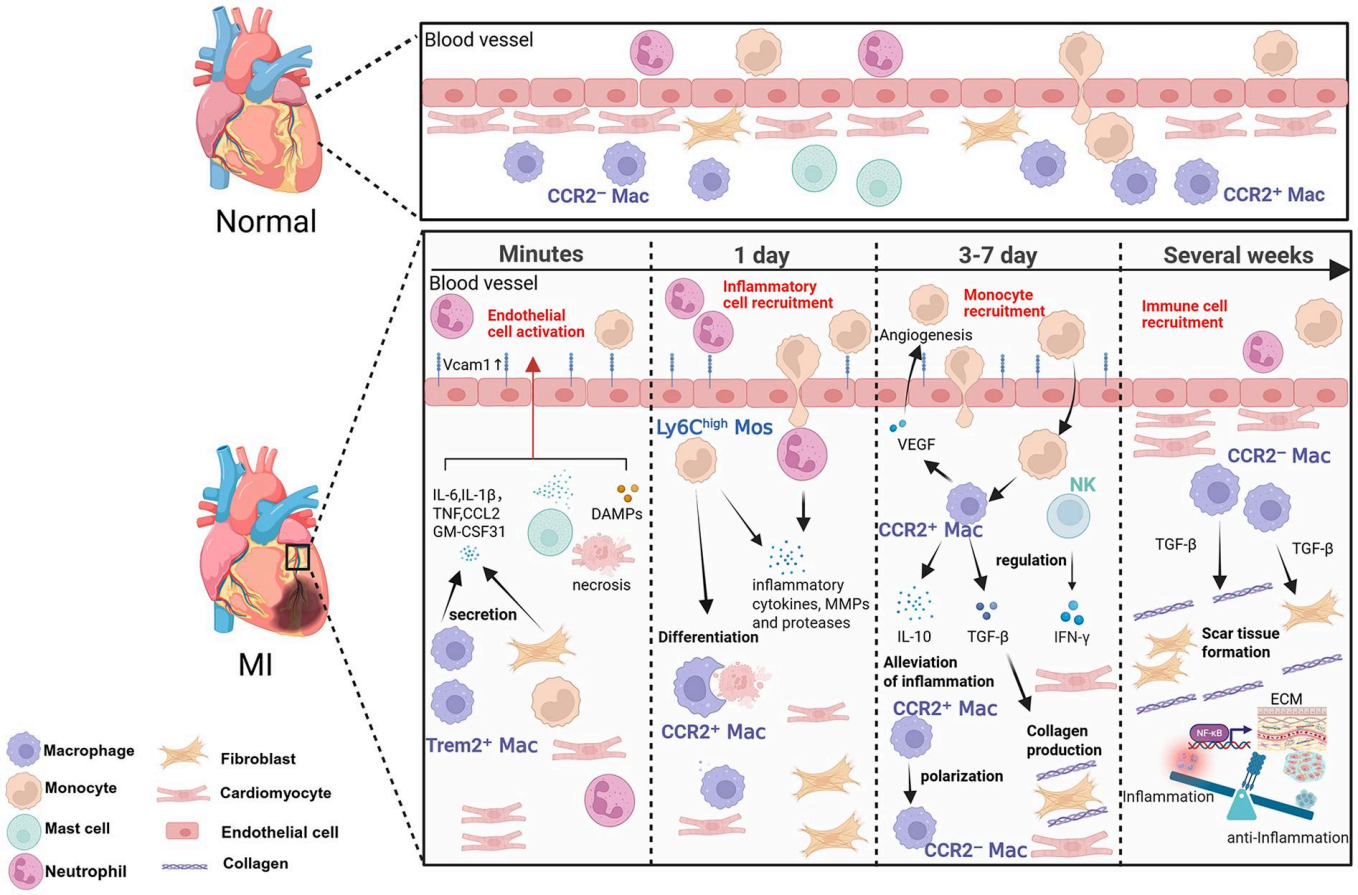
Response of immune cells and mediators following myocardial infarction. Five phases are shown from left to right: normal, minutes, day 1, days 3-7, and several weeks post-MI. Cell types include endothelial cells, mast cells, tissue-resident macrophages (Trem2⁺, CCR2^−^), neutrophils, monocytes (Ly6C^high^), CCR2⁺ pro-inflammatory macrophages, CCR2^−^ reparative macrophages, fibroblasts, NK cells, and Tregs. Arrows and labels indicate key events: differentiation, pro-inflammatory signaling, and reparative/resolution processes. Key molecules: DAMPs, IL-1β, IL-6, TNF, CCL2, TGF-*β*, IL-10, VEGF. This figure integrates the single-cell-based functional state framework described in the text. Created in BioRender. Huang, L. (2026) https://BioRender.com/lvpw9fo.

MI triggers a potent sterile inflammatory response, ultimately causing tissue injury and the loss of cardiomyocytes in the myocardial region supplied by the obstructed vessel (26). This activation triggers a meticulously orchestrated inflammatory cascade, essential for clearing necrotic debris and facilitating tissue repair (27). However, the immune response in MI is a double-edged sword (28). An effective and timely response is essential for effective wound healing and scar formation, whereas excessive, prolonged, or dysregulated inflammation can exacerbate initial injury, impair contraction, and promote adverse ventricular remodeling, ultimately leading to heart failure. Thus, the balance between pro-inflammatory and pro-resolving signals determines the trajectory of post-infarct cardiac recovery. Despite established roles of inflammatory and immune pathways in ischemic heart disease, the functional contributions of individual immune cell subsets remain incompletely understood. Conventional cell-type labels are insufficient to capture post-MI immune heterogeneity. Single-cell sequencing(scRNA-seq) has instead defined a spectrum of functional states: e.g., Trem2^+^ lipid-associated macrophages (phagocytic, non-inflammatory), Mrc1^+^ repair macrophages, and Il1b^+^ inflammatory monocytes (29, 30). These states interconvert via transitional intermediates, challenging binary M1/M2 or Ly6C^high/low^ classifications. Accordingly, throughout this review we adopt a state-based framework, referring to functional programs rather than static phenotypes, which directly informs the immunomodulatory strategies discussed later. This part delineates the immune mechanisms underpinning ischemic heart disease, focusing on MI and the subsequent myocardial repair process (Figure 1).

### 2.1 Normal (steady state)

In the healthy heart, resident macrophages (including CCR2^−^subsets) maintain immune surveillance and tissue homeostasis. Mast cells and a sparse population of other immune cells are present but quiescent. Endothelial cells express low levels of adhesion molecules, and no significant inflammatory signal is detected.

### 2.2 Minutes after ischemia: tissue-resident macrophages and DAMPs

Within minutes after ischemia, a complex cascade of coordinated cellular events is initiated within the heart tissue and vasculature. Tissue-resident macrophages, integral components of the innate immune compartment, promptly detect ischemic injury. The spatial transcriptomics and scRNA-seq analyses of MI-related immune cell subsets have delineated their spatiotemporal dynamics, identifying a macrophage subset with anti-inflammatory properties (Trem2^hi^) prevalent in infarcted hearts (29). A novel cardiac macrophage subset, termed Bhlhe41^+^ macrophages in infarction zones, has been shown to limit fibrosis, highlighting their dynamic roles in cardiac pathology and repair (31). Concurrently, cardiomyocytes, the cardiac muscle cells directly compromised by the lack of perfusion, also mount a stress response. Macrophages and cardiomyocytes cooperatively elicit diverse inflammatory mediators, notably cytokines (IL-1β, IL-6, TNF-α) and chemokines (CCL2) (32, 33). These molecules are crucial for amplifying the inflammatory signal within the local cardiac environment. Dying cardiomyocytes release DAMPs, cardiac fibroblasts secrete hematopoietic growth factors such as granulocyte-macrophage colony-stimulating factor, mast cells degranulate, and endothelial cells undergo activation (34, 35). Concurrently, neutrophils and monocytes undergo mass mobilization predominantly derived from bone marrow hematopoietic stem and progenitor cells post-MI (36), with minor contributions from extramedullary reservoirs like the spleen. These recruited leukocyte actively participate in the developing inflammatory response. An increase in circulating inflammatory leukocytes thereby enhances their infiltration into the myocardium.

### 2.3 Day 1: neutrophil infiltration and debris clearance

On the first day after infarction, these immune cells primarily clear necrotic debris, including apoptotic and necrotic cardiomyocytes. This clearance is mediated through specific molecular pathways involving several scavenger receptors, notably the tyrosine-protein kinase MER. Additionally, immune cells facilitate the removal of cardiomyocyte-derived DNA via interferon regulatory factor 3 (IRF3)-dependent mechanisms (37). Following MI, activated neutrophils infiltrate the infarcted heart and release pre-formed mediators via degranulation and neutrophil extracellular traps formation (38). These processes release molecules like pentraxin 3 (PTX3), myeloperoxidase, and other bactericidal proteins. Notably, PTX3, which is stored in neutrophil-specific granules and released upon activation, localizes within the structure of neutrophil extracellular traps (39). Beyond being a biomarker, PTX3 appears to play a cardioprotective role by modulating inflammation, regulating complement activation, and limiting excessive neutrophil recruitment to the injured myocardium, thereby potentially influencing the extent of tissue damage and repair (40). For example, their interaction with leukocytes modulates leukocyte behavior, enhancing migration to the injury site and immunological functions, thereby amplifying the inflammatory cascade (41). Furthermore, interactions with vascular endothelial cells can alter endothelial phenotype and increase vascular permeability, facilitating extravasation of immune cells and inflammatory mediators into surrounding tissues to promote inflammation. Additionally, interactions with cardiomyocytes can disrupt cardiomyocyte function and propagate the inflammatory state within the cardiac tissue, potentially influencing cardiac pathophysiologyn (42).

### 2.4 Days 3–7: resolution phase and macrophage phenotypic switch

By day three post-MI, prototypical pro-inflammatory mediators such as TNF-α and IL-6 begin to decline, signifying the resolution phase and attenuation of the early inflammatory cascade. These cytokines are essential for immune cell recruitment, vascular permeability enhancement, and immune activation. Their reduction occurs through negative feedback mechanisms wherein anti-inflammatory mediators suppress pro-inflammatory cytokine production. Concurrently, elevated levels of IL-10 and TGF-*β* mark the transition from acute to chronic inflammation. During this transition, neutrophils are progressively replaced by reparative cells, including macrophages and fibroblasts. Neutrophil numbers decline significantly by day three and are largely resolved by day seven. Meanwhile, monocytes progressively accumulate and differentiate into tissue macrophages exhibiting a reparative phenotype, which release IL-10 and vascular endothelial growth factor (VEGF) (43, 44). These mediators perform critical functions: IL-10 collaborates with Tregs to suppress inflammation; TGF-*β* promotes fibroblast-mediated collagen production; and VEGF drives angiogenesis (45). Natural killer (NK) cells are also activated after MI, contributing to early inflammation through cytokine production (e.g., IFN-*γ*) and cytotoxicity, while emerging evidence suggests a regulatory role in limiting excessive inflammation (46). While the acute inflammatory phase focuses on eliminating inflammatory triggers and damage containment, the chronic phase emphasizes tissue restoration. Collectively, cytokine dynamics govern the body's capacity for homeostatic restoration and post-injury recovery, representing a pivotal regulatory mechanism in inflammatory resolution and tissue repair processes.

### 2.4 Several weeks: reparative phase and scar maturation

Over subsequent weeks, the dynamic interplay between innate immune components (neutrophils, monocytes, mast cells) and adaptive immune elements (lymphocyte subsets) progressively diminishes (47). The transition to the reparative phase is characterized by neutrophil decline and the emergence of Ly6C^low^ macrophages. This results in a reduction of overall immune cell populations within the heart, eventually returning to a steady-state level. Experimental researches have demonstrated that Excessive recruitment of immune cells during the initial acute stage may lead to impaired healing and adverse ventricular remodeling, including infarct zone thinning (48).

## 3 Mechanism of crosstalk between heart and immune organs after MI

**Figure 2:**
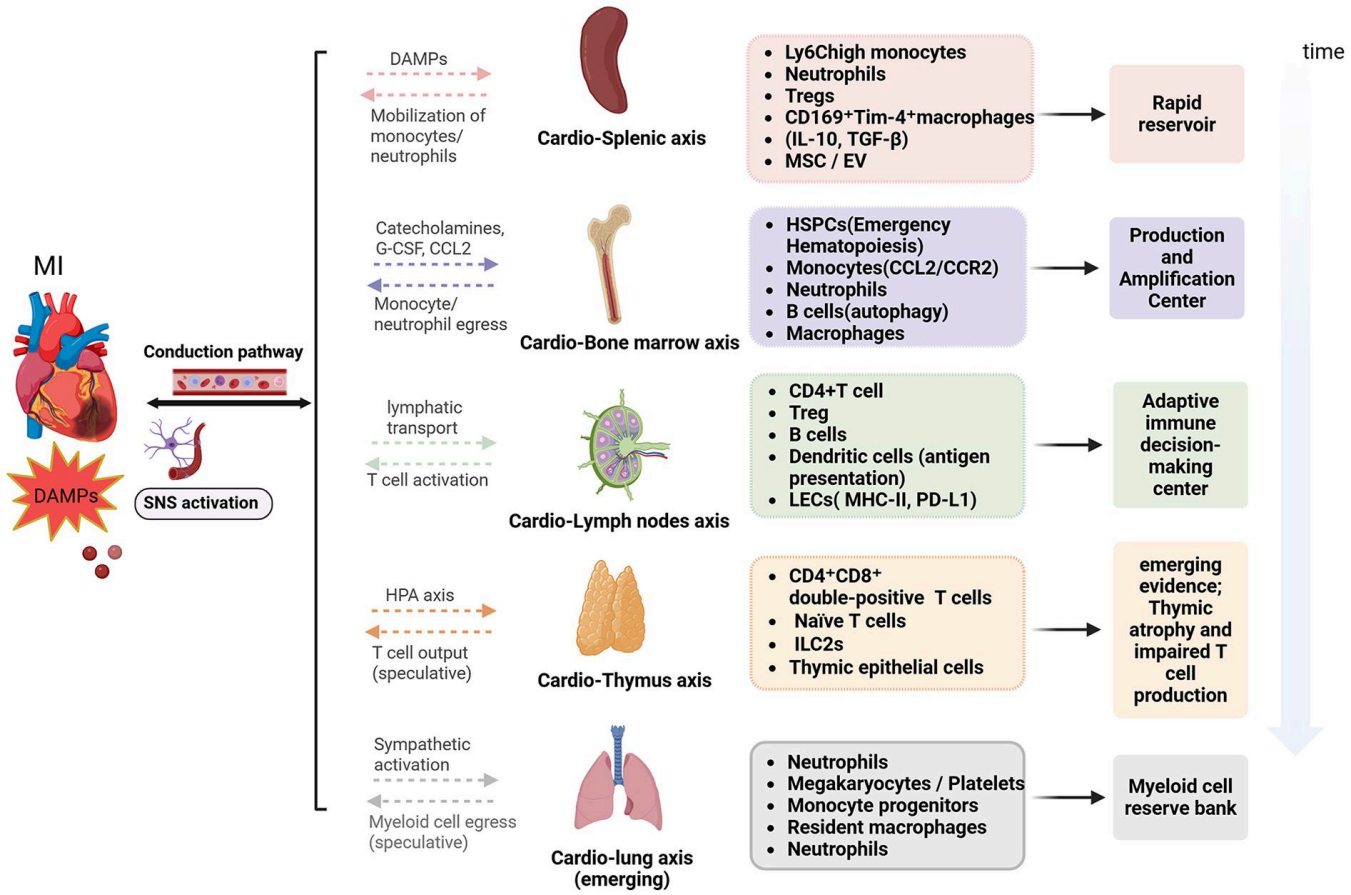
Cellular components of heart-immune organ axes after myocardial infarction. The infarcted heart communicates with five major immune organs via distinct signaling pathways. Colored dashed boxes list the key immune cell types involved in each axis, based on the detailed descriptions in Sections 3.1–3.5. Arrows indicate the direction of signaling (e.g., DAMPs, cytokines, catecholamines, neural signals). HSPCs, hematopoietic stem/progenitor cells; Tregs, regulatory T cells; HPA, hypothalamu-pituitary-adrenal. The cardio-thymus axis remains partially speculative and requires further validation(Section 3.5). The cardio-lung axis, listed cell types are based on emerging evidence (Section 3.5). Created in BioRender. Huang, L. (2026) https://BioRender.com/8ut0krz.

The crosstalk between the heart and immune organs entails complex cellular signaling and molecular mechanisms following MI. Ischemic injury triggers liberation of endogenous danger signals, particularly classical DAMPs such as high mobility group box 1 (HMGB1) and heat shock proteins (HSPs). These molecules activate innate immune responses through pattern recognition receptors, orchestrating an inflammatory cascade that recruits and activates immune cells at the injury site. Distinct immune cell populations interact through cytokine networks during MI. Macrophages release mediators including IL-6 and TNF-α, stimulating additional immune cells and establishing intercellular signaling networks. Extracellular vesicles facilitate communication through inflammatory cytokines, miRNAs, and other molecular mediators. Coordinated intercellular communication between diverse cardiac cell populations including cardiomyocytes, fibroblasts, and endothelial cells is essential for maintaining homeostasis after cardiac injury (49–51). The coordinated activity of both primary (bone marrow, thymus) and secondary (spleen, lymph nodes) lymphoid organs critically shapes the inflammatory response after MI. In the following section, we subsequently summarize current research elucidating heart-immune organ interaction mechanisms, as shown in Figure 2.

### 3.1 The cardio-splenic axis

AMI triggers cardiomyocyte necrosis and the release of large amounts of damage-associated DAMPs, initiating both local and systemic inflammatory responses. As a key secondary lymphoid organ, the spleen serves as a major reservoir of immune cells, including monocytes and neutrophils, and functions as a rapid-response pool during systemic stress (52, 53). Following MI, the response of monocytes/macrophages can be divided into two main phases: an early pro-inflammatory phase and subsequent repair phase. In the initial stage of infarction (peaking around day 3), classical Ly6C^high^ monocytes are recruited in large numbers and differentiate into pro-inflammatory macrophages resembling the M1 phenotype. These cells secrete inflammatory mediators, clear necrotic tissue and apoptotic cells, and create conditions for subsequent repair. During this period, macrophage metabolism shifts toward glycolysis to adapt to the hypoxic environment. Subsequently, their pro-inflammatory activity is gradually downregulated while phagocytic function is enhanced, marking the transition toward the repair phase. Around day 7, CD169⁺ Tim-4⁺ macrophages in the splenic marginal zone serve as central regulators of the heart-spleen axis. Positioned at critical entry sites for blood into the white pulp, they mediate the phagocytosis of apoptotic cells and damage-associated material via Tim-4, thereby initiating an immunomodulatory program: secreting anti-inflammatory factors such as IL-10 and TGF-*β*, suppressing the expression of pro-inflammatory cytokines, and promoting Treg differentiation. Meanwhile, Ly6C^low^ monocytes are recruited in smaller numbers and differentiate into anti-inflammatory macrophages resembling the M2 phenotype, which promote angiogenesis, cell proliferation, and extracellular matrix synthesis to drive tissue repair. Some macrophages also express fibroblast-related genes such as collagen, directly contributing to scar formation. These two waves of monocyte/macrophage dynamics coordinate the inflammatory response with the repair process, exerting a crucial influence on tissue outcome after myocardial infarction (54–56).

Liu et al. found that in a model of ischemic cardiomyopathy, intravenous administration of mesenchymal stem cells (MSCs) attenuated the progressive deterioration of left ventricular function and adverse remodeling in mice, leading to improved left ventricular performance. These effects were partly mediated by systemic anti-inflammatory actions. Moreover, the number of MSCs detected in the spleen of these mice was significantly higher than that in neonatal mice, suggesting that myocardial injury generates systemic signals that drive the heart-spleen axis (57). Study have demonstrated that in a preclinical mouse model of experimentally-induced MI/RI, the administration of bone marrow-derived mesenchymal stem cells (BM-MSCs) effectively alleviated MI/RI in mice by inducing Tregs in the spleen and upregulating IL-10 and TGF-*β*. This suggests that the spleen serves as a critical organ in which transfused BM-MSCs cultivate Tregs and promote immunomodulation, thereby mitigating MI/RI (58). Akerba et al. found that extracellular vesicles (EVs) released from plasma activate signals for monocyte mobilization and transcriptional activation in the spleen after AMI, revealing the mechanism by which ischemic myocardium transmits both monocyte mobilization and transcriptional activation signals following MI (59). Xu et al. found that in the presence of myocardial injury, intravenous or intramyocardial injection of MSC-EV resulted in the highest and most effective delivery of MSC-EV in the spleen and liver, thereby contributing to enhanced myocardial protection (60).

In summary, the cardio-splenic axis acts as a critical bridge connecting cardiac injury and systemic immune regulation. CD169^+^ Tim-4^+^ macrophages in the splenic marginal zone serve as a central processing unit, coordinating the transmission of reparative signals. The therapeutic effects of MSCs/EVs essentially involve reprogramming the splenic immune function, thereby mobilizing a systemic immunomodulatory network to achieve remote cardioprotection. This indicates a shift in their mechanism of action from the traditional “cell replacement” paradigm toward a new paradigm centered on systemic immune reprogramming. Thus, targeting the cardio-splenic axis represents promising therapeutic strategy for improving post-MI recovery. Future research should focus on developing precise delivery systems to the splenic marginal zone, optimizing the timing and dosage of immunomodulatory therapies, and identifying specific molecular targets within the splenic macrophage population to enhance reparative signaling while minimizing off-target effects.

### 3.2 The cardio-bone marrow axis

MI, as an acute ischemic event, involves a pathophysiological process that extends far beyond localized myocardial necrosis, representing instead a complex disease entailing a systemic response. Recent studies have revealed that during the occurrence and progression of MI, there exists a dynamic and bidirectional “Cardio-Bone marrow axis”, which closely links the remote hematopoietic organs of the bone marrow to the damaged heart, collectively regulating the entire course of inflammatory response, tissue repair, and remodeling (61). AMI leads to necrosis of cardiomyocytes, releasing large amounts of DAMPs. These signals are transmitted throughout the body via the circulatory system. The bone marrow, as the primary reservoir for hematopoiesis and immune cells, serves as a critical response hub. Excessive activation of cardiac sympathetic nerves and the release of catecholamines can directly act on adrenergic receptors on the surface of hematopoietic stem and progenitor cells in the bone marrow, rapidly mobilizing neutrophils and monocytes into the circulation (62). Meanwhile, local inflammatory cells (such as activated platelets and monocytes) in the heart, along with the ischemic tissue itself, produce and release a large number of cytokines and chemokines, among which CCL2, IL-1β, and IL-6 act as key messengers. These factors act systemically on the bone marrow microenvironment, reshape hematopoietic homeostasis, and induce an“emergency hematopoiesis”program. This process shifts the lineage bias of the bone marrow from steady-state lymphopoiesis toward the massive production of granulocytes and monocytes. Newly generated monocytes, especially those strongly mobilized via the CCL2-CCR2 axis, enter the bloodstream and migrate to the infarcted myocardium. Notably, the spleen also plays a significant role in this axis, serving as an additional reservoir of monocytes that similarly responds to these remote signals and contributes inflammatory cells (63).

Studies have confirmed that MI can induce systemic neuroendocrine activation, which subsequently mediates glucocorticoid-dependent autophagic death of bone marrow-resident naïve B lymphocytes via Na^+^/H^+^ exchanger 1 (64). Intriguingly, empagliflozin can exert cardioprotective effects by reversing this autophagic process, revealing a novel mechanism of adaptive immune regulation in myocardial repair. On the other hand, deficiency of calpain small subunit 1 in bone marrow-derived macrophages alleviates myocardial injury by maintaining mitochondrial homeostasis and suppressing the NLRP3 inflammasome signaling pathway (65). Recent studies have further revealed that in diabetic MI models, the heart-spleen-bone marrow axis exhibits pathological dysregulation, characterized by delayed immune responses and impaired differentiation of endothelial progenitor cells (66). Thus, the sustained or dysregulated activation of this axis exhibits a double-edged sword effect. Following AMI, a chronic, low-grade state of bone marrow activation may lead to persistent systemic and cardiac-localized inflammation, which is associated with the progression of atherosclerosis, increased plaque instability, and distal organ injury (67). Moreover, this systemic inflammatory milieu can accelerate the clonal evolution of hematopoietic stem cells in the bone marrow, potentially leading to the acquisition of somatic mutations linked to aging or atherosclerosis. This, in turn, further drives the production of inflammatory cells in a feedforward manner, creating a vicious cycle. This mechanism is considered one of the key contributors to the persistently elevated long-term cardiovascular risk in post-MI patients.

Therefore, the cardio-Bone marrow axis constitutes the core component of the systemic response to myocardial infarction, transforming the initial local cardiac injury into a sustained systemic pathological process that profoundly influences both acute-phase outcomes and long-term repair and remodeling. A deeper understanding of the fine regulatory mechanisms of this axis will provide a crucial theoretical foundation for developing novel therapeutic strategies aimed at targeting systemic inflammation, interrupting the vicious cycle, and thereby improving the prognosis of patients after myocardial infarction.

### 3.3 The cardio-lymph nodes axis

Lymph nodes (LNs) are critical immune hubs, but their roles are site-dependent. The mediastinal LNs (mLNs), which directly drain the heart, are the most relevant; peripheral LNs receive only systemic signals. After MI, cardiac self-antigens (e.g., myosin) are transported via lymphatics to mLNs, where dendritic cells present them to T cells, potentially breaking immune tolerance. Lymphatic endothelial cells (LECs) actively regulate this process by expressing MHC-II and co-inhibitory molecules (e.g., PD-L1), though their post-MI role is understudied.

Within LNs, Tregs suppress inflammation, effector T cells produce cytokines, and B cell depletion improves cardiac function (68). LN-derived exosomes attenuate injury via PTEN-PI3K/AKT signaling (69). LN T cell activity correlates with functional recovery (70). Modulating intranodal responses (e.g., targeting antigen presentation or LEC function) is a promising strategy, and LN status may serve as a biomarker.

### 3.4 Emerging evidence for a cardio-thymus axis

Compared with the well-established bone marrow–spleen axes, the role of the thymus in post-MI immune modulation remains less defined and largely exploratory. As a primary lymphoid organ, the thymus drives T cell ontogeny and maturation, and could theoretically influence cardiac repair by shaping the T cell repertoire. However, direct mechanistic evidence for a heart–thymus crosstalk after MI is still limited.

Several indirect observations suggest potential links. Ischemia-induced thymic atrophy and impaired lymphopoiesis have been reported following MI, reflected by decreased thymic index and reduced T cell counts (71). A regulatory network involving miR-15b/miR-29a modulates VEGF signaling within thymic adipose tissue in elderly ischemic cardiomyopathy patients, hinting at diagnostic possibilities (72). Additionally, a neuroimmune-endocrine network coordinating heart–thymus crosstalk has been proposed, with emerging evidence specifically implicating activation of the hypothalamus-pituitary-adrenal (HPA) axis as a mediator of post-MI thymic injury (73, 74). Eosinophils and thymus-resident type 2 innate lymphoid cells have also been implicated, and proteomic analyses associate thymosin *β*4 levels with first MI risk (75).

Nevertheless, these findings remain correlative, and key questions persist: how cardiac ischemic injury modifies thymic architecture, whether thymus-derived T cells functionally contribute to post-MI repair vs. maladaptive inflammation, and if thymic involution is a cause or consequence of systemic immune dysregulation. Until these are addressed, the cardio-thymus axis should be considered a speculative but promising frontier rather than a validated pathway. We highlight it here to encourage future investigation, but caution against assigning it equal weight to the bone marrow or spleen axes at present.

### 3.5 The circulatory conduit and the lung as an emerging interface

Beyond individual immune organs, the circulatory system serves as the essential physical conduit for heart-immune crosstalk. Virtually all soluble mediators (cytokines, DAMPs, hormones), extracellular vesicles, and immune cell trafficking between the heart and remote organs transit through the blood. The composition of circulating immune cells, particularly the dynamic balance of classical/intermediate/non-classical monocytes, neutrophil subsets, and T cell populations, which directly reflects and influences post-MI systemic immunity (76). Thus, the blood axis is not a separate organ but the integrative fluid compartment that enables all other axes; its analysis provides accessible biomarkers and potential therapeutic targets (e.g., leukapheresis, cell-based therapies).

The lung, in contrast, is increasingly recognized as an active participant. Beyond its role as a filter, the lung harbors a large pool of resident and recirculating immune cells, including megakaryocytes that produce platelets and monocyte progenitors (77). After MI, lung inflammation can be triggered by sympathetic activation and circulating DAMPs, and the lung may serve as a reservoir for myeloid cells that subsequently migrate to the heart (78). However, direct evidence for a dedicated “cardio-pulmonar*y* axis” remains preliminary, and we present it as a promising but incompletely defined frontier.

### 3.6 Integration with the heart-immune axis framework

Here, we contextualize the organ-specific findings within the proposed integrated framework of the heart-immune axis. The observed rapid egress of monocytes and neutrophils from the spleen within the first 24 h post-MI underscores its role as the primary reservoir for rapid innate immune deployment (79). The immediate response is likely attributable to DAMPs signaling and acute sympathetic nervous system (SNS) activation, which positions the spleen as the primary responder in this axis (80). Subsequently, the expansion of myeloid progenitors and sustained output of inflammatory monocytes from the bone marrow, peaking at days 3–7, designate it as the central hub for prolonged production and amplification of the systemic inflammatory response (81). This phase aligns with and is likely fueled by elevated systemic levels of cytokines such as GM-CSF and IL-6. While the thymus exhibited a delayed reduction in cellularity, its potential role may lie in long-term immunomodulation, possibly influencing T-cell repertoire and cardiac remodeling in the chronic phase. Finally, the observed antigen presentation and T-cell activation in the mediastinal lymph nodes highlight their critical function as the key decision-making center for adaptive immunity. This node acts as an integrative site, processing cardiac-derived antigens to potentially shape protective or detrimental immune responses that influence late repair and fibrosis. Collectively, these sequential and organ-specialized events, including rapid splenic mobilization, sustained bone marrow production, and lymphoid organ instruction, dynamically constitute the operational cascade of the heart-immune axis following MI.

## 4 Immunomodulation-based therapeutic strategies for MI: mechanistic insights, clinical progress, and translational challenges

The conventional management of AMI centers on reperfusion therapy. However, the revascularization process itself can induce MI/RI, in which the excessive activation of the immune-inflammatory response is a pivotal mechanism leading to secondary cardiomyocyte damage, infarct expansion, and adverse ventricular remodeling (82). Consequently, precisely modulating the immune response to mitigate reperfusion injury, protect myocardial tissue, and improve long-term outcomes has emerged as a significant research direction in cardiovascular medicine (Table 3). Given the functional state diversity revealed by scRNA-seq, successful immunomodulation will likely require targeting state-specific markers (e.g., TREM2, SPP1) rather than depleting entire lineages. Nevertheless, significant challenges remain in translating these findings into clinical applications.

**Table 3:** Developmental stages and key outcomes of immunomodulatory strategies for MI.

Strategy	Mechanism/Target	Trial/phase	Key outcome/status
Canakinumab	Anti-IL-1β	CANTOS (III)	Positive: reduced recurrent CV events; improved infection risk
Tocilizumab	Anti-IL-6R	Multiple (II/III)	Mixed efficacy; long-term safety unclear
Pexelizumab	Anti-C5	COMMA, COMPLY (III)	Failed—no improvement in mortality/infarct size
Abatacept	CTLA-4-Ig	Preclinical/observational	Promising; no RCT
Treg therapy	Adoptive Tregs	Early clinical	Not tested in MI; manufacturing/logistical hurdles
Colchicine	Neutrophil inhibition	COLCOT, LoDoCo2 (III)	Positive; now in clinical practice for secondary prevention
Statins	Pleiotropic anti-inflammatory	Multiple large trials	Positive; standard of care
MSCs/EVs	Paracrine	Pre/Phase I/II	Regenerative potential; needs larger trials
RIC	Neuro-immune	Pre/small clinical	Mixed results; mechanism not fully defined
ZBP1 inhibitor	PANoptosis block	Preclinical	Effective; human translation pending

RIC, remote ischemic conditioning. “Failed” indicates no benefit on primary endpoints. “Positive” means significant improvement in cardiovascular outcomes. For original references, see Section 4.1–4.3.

### 4.1 Therapeutic strategies targeting the innate immune system

Strategies targeting the innate immune system primarily encompass the regulation of inflammatory cells and interventions against inflammatory cytokines. The dual role of macrophages in cardiac damage and repair accentuates their therapeutic potential (83). Specifically, therapeutic strategies that shift the balance from CCR2^+^ pro-inflammatory macrophages toward TREM2^+^ reparative subsets [Preclinical; CCR2^+^ → TREM2⁺macrophages; cardio-splenic axis] or drive macrophage repolarization from the M1 to the M2 phenotype via pathways such as PD-1/STAT6 [Preclinical; M1→M2 macrophages; cardio-splenic axis] have demonstrated efficacy in ameliorating excessive inflammation and promoting tissue repair (84).

In cytokine-targeted therapy, IL-1 inhibitors such as canakinumab [Phase III; IL-1β; cardio-bone marrow axis] (85), validated by the landmark CANTOS trial, significantly reduce the risk of recurrent cardiovascular events in MI patients with elevated high-sensitivity C-reactive protein, establishing an evidence-based foundation for cardiovascular anti-inflammatory therapy (86, 87). Although IL-6 inhibitors (e.g., tocilizumab) [Phase II/III; IL-6R; systemic] initially demonstrate anti-inflammatory potential, their long-term efficacy and safety require further validation (88, 89). In contrast, the application of TNF-α inhibitors [Clinical trials; negative; TNF-*α*] has been limited due to early studies suggesting a potential exacerbation of heart failure (90).

Regarding the regulation of inflammatory cells, although complement inhibitors like pexelizumab [Failed Phase III; complement C5; innate] have not yielded definitive benefits in clinical trials, related mechanistic investigations continue to deepen. Indeed, the translation of many innate immune targets has been challenging. For instance, while preclinical studies strongly supported the role of complement cascade in MI/RI, large-scale clinical trials (e.g., COMMA, COMPLY) with pexelizumab failed to demonstrate significant improvement in primary clinical endpoints, highlighting the complexity of targeting this system in humans and potential discrepancies between animal models and clinical disease (91). Concurrently, novel drug development is focusing on inhibiting the chemotaxis and migration of neutrophils to the infarcted myocardium [Preclinical; neutrophil chemotaxis; cardio-bone marrow axis] to alleviate early inflammatory damage. However, broad inhibition of neutrophils carries risks of impaired host defense, underscoring the need for precisely timed or spatially restricted interventions.

### 4.2 Precision modulation of the adaptive immune system

Current strategies for precise modulation of the adaptive immune system after MI primarily target key T-cell activation pathways and Tregs (92). On one hand, CTLA-4, a critical immune checkpoint molecule, delivers inhibitory signals to prevent excessive T cell activation. Conversely, immune checkpoint inhibitors targeting CTLA-4 have been shown to exacerbate myocardial inflammation and promote heart failure, underscoring the physiological importance of CTLA-4–mediated inhibition in preventing immune overactivation (93). The CTLA-4-Ig fusion protein (abatacept) mimics the function of CTLA-4, competitively binding B7 molecules on antigen-presenting cells, thereby blocking the CD28-B7 co-stimulatory pathway and inhibiting T cell overactivation. Following MI, autoantigens released from necrotic cardiomyocytes trigger aberrant immune responses, exacerbating inflammation, expanding the infarct size, and promoting adverse cardiac remodeling, ultimately leading to heart failure. Experimental evidence from a mouse model of cardiac ischemia-reperfusion injury demonstrates that treatment with CTLA-4-Ig (abatacept) significantly preserves cardiac function by nearly completely inhibiting the robust CD4^+^ T cell response in the heart and reducing innate inflammatory cell infiltration. [Preclinical/observational; CD28-B7; cardio-splenic axis] While clinical observational data suggest potential improvement in MI outcomes for relevant patients, its efficacy in AMI still requires validation through randomized controlled trials (RCTs) (94, 95).

On the other hand, Tregs are a naturally immunosuppressive T cell subset that highly expresses CTLA-4. Tregs can directly “trans-endocytose” or otherwise modulate B7 molecules from antigen-presenting cells via their surface CTLA-4, or employ other mechanisms to suppress effector T cell activity. Isolating Tregs from patients, expanding and activating them *ex vivo*, and subsequently reinfusing them enables these cells to home to the site of cardiac injury. There, through multiple mechanisms including their highly expressed CTLA-4, they locally suppress excessive immune responses and promote a reparative environment. [Early clinical (other inflammatory diseases);Tregs; cardio-splenic axis] These two classes of strategies collectively advance the development of immunomodulatory therapies towards precision and personalization post-MI (96, 97).

However, clinical translation faces distinct challenges. For CTLA-4-Ig (abatacept), while preclinical data are compelling, its potent systemic immunosuppressive effect raises concerns about increased infection risk in AMI patients, a population often with comorbidities. The absence of large-scale RCTs leaves its risk–benefit profile unclear in acute settings (94). Similarly, adoptive Treg therapy, though highly precise, confronts practical challenges including complex and costly *ex vivo* expansion processes, variability in Treg stability and function after reinfusion, and the need for stringent patient stratification. Early-phase clinical trials in other inflammatory conditions have shown mixed results regarding Treg persistence and efficacy, indicating a non-trivial path towards cardiovascular applications. This highlights T cell costimulation as a central driver of post-MI damage and positions CTLA-4-Ig therapy as a promising yet clinically unproven strategy for clinical translation to mitigate heart injury after an ischemic event.

### 4.3 Clinical progress in other immunomodulatory strategies

Beyond strategies targeting specific immune pathways, several therapies with immunomodulatory functions have achieved significant clinical progress. Among these, colchicine, supported by the COLCOT and LoDoCo2 trials, has demonstrated that its low-dose regimen significantly reduces cardiovascular event risks [Phase III/clinical practice; neutrophil; systemic] (98–100). Due to its oral convenience and cost-effectiveness, it has become an important option for the secondary prevention of atherosclerotic cardiovascular disease. Statins, owing to their pleiotropic effects including anti-inflammatory actions beyond lipid-lowering, have gained widespread consensus in cardiovascular protection [Clinical practice; pleiotropic; multi-axis] (101, 102). In novel therapeutic avenues, MSCs therapy modulate the immune microenvironment via paracrine mechanisms, promoting macrophage polarization towards a reparative phenotype and showing regenerative potential [Preclinical/Phase I/II; macrophages/Tregs; cardio-splenic axis] (103). Remote ischemic conditioning, a non-invasive technique, appears to elicit systemic protective responses that indirectly temper detrimental inflammation [Preclinical/early clinical; neuro-immune; multiple axes] (104). Furthermore, upstream intervention in cardiomyocyte death has emerged as a crucial strategy. Inhibiting ZBP1 - a key driver of PAN optosis (a synergistic cell death pathway) - with a specific small-molecule inhibitor, significantly mitigated MI/RI by disrupting the formation of the ZBP1/RIPK3 complex [Preclinical; cardiomyocyte PANoptosis; not immune-cell-directed] (105). These strategies collectively enrich the therapeutic spectrum for immunomodulation after MI.

### 4.4 Translational challenges and future research

The translation of mechanistic insights into clinical success for MI immunomodulation faces significant challenges. Despite strong preclinical rationale, many agents have demonstrated neutral or negative outcomes in pivotal trials. These setbacks stem from several interrelated factors. First, inflammation is dynamic and dual-natured: immune components such as macrophages or IL-6 can exert both harmful and reparative effects at different stages after MI, rendering intervention timing crucial. Broad suppression may thus disrupt necessary healing processes (106). Second, patient heterogeneity-including differences in baseline inflammation, comorbidities such as diabetes or autoimmune conditions, and genetic background-likely shapes therapeutic response. This is illustrated in the CANTOS trial, where clinical benefit was largely confined to patients with higher inflammatory burden (86). Sex differences represent another critical dimension of patient heterogeneity. Premenopausal females are relatively protected against MI due to estrogen-mediated immune modulation, including M2 macrophage polarization and reduced neutrophil infiltration (107). Conversely, females exhibit more pronounced autoimmune activation post-MI, with higher levels of autoantibodies and pro-inflammatory cytokines (108). Most preclinical models use young males, limiting translational relevance. Third, conventional animal models have limitations; standard rodent MI models often fail to fully replicate human atherosclerosis, comorbidities, and immune complexity. Fourth, the failure of pexelizumab (anti-C5) provides a cautionary example of incomplete mechanistic validation. Although preclinical studies showed robust complement inhibition and infarct reduction, an APEX-AMI substudy found that pexelizumab blocked C5a *in vitro* but failed to suppress terminal complement complex (sC5b-9) in patients, likely due to complement activation preceding drug administration. This timing mismatch and the lack of downstream biomarker modulation illustrate how inadequate understanding of pathway dynamics can derail translation despite strong preclinical rationale (109). Finally, many early-phase trials have relied on surrogate endpoints such as infarct size reduction, which have not consistently predicted improvements in clinically meaningful outcomes like heart failure hospitalization or cardiovascular death in later-phase studies.

Future research should therefore focus on stage-specific and personalized immunomodulation. The ultimate aim is to shift from nonspecific immunosuppression toward precision immunomodulation: a finely tuned strategy that suppresses harmful inflammation while preserving innate healing, thereby providing lasting cardioprotection and better long-term outcomes.

## 5 Discussion

The emerging discipline of cardioimmunology has profoundly transformed our perception of the cardiac response following MI, establishing the immune system as a pivotal regulator of both tissue damage and repair. Although a tightly coordinated immune response is recognized as crucial for clearing cellular debris, stabilizing the extracellular matrix, and promoting regenerative processes, the underlying mechanisms that determine its transition from a beneficial role to a state of detrimental chronic inflammation remain immensely complex (110). The“heart-immune axis” represents not merely a set of parallel pathways, but rather a dynamic and hierarchically organized network. This system is triggered by cardiac injury and proceeds to engage the spleen, bone marrow, thymus, and lymph nodes in a distinct temporal sequence, thereby coordinating, amplifying, and ultimately resolving the immune response. Notably, this process undergoes continuous temporal evolution, indicating that immunomodulatory interventions may yield differing effects depending on the stage of post-MI healing, whether in the acute, subacute, or chronic heart failure phase. The equilibrium is strongly influenced by specific immune cell subsets whose functional plasticity presents not only therapeutic potential but also considerable scientific debate. However, the functional duality of these immune cells, coupled with discrepancies in experimental findings, underscores the complexity of the heart-immune axis.

The functional duality of macrophages underscores the complex immune response in cardiac injury. While resident macrophages promote repair via efferocytosis—the clearance of apoptotic cardiomyocytes, which prevents secondary necrosis and promotes inflammation resolution, infiltrating CCR2^+^ macrophages drive inflammation via TNF-α and IL-1β. Key molecular players include MerTK (receptor for apoptotic cell uptake), TIM-4 (phosphatidylserine tethering), ABCA1 (cholesterol efflux), and CD47 (a “don't eat me” signal). Beyond clearance function, the survival of tissue-resident cardiac macrophages themselves is critical. Recent work shows that SerpinB2 regulates mitochondrial oxidative phosphorylation and prevents cytochrome c release via antioxidant glutathione production, thereby promoting tissue-resident macrophage survival (111). However, the M1/M2 classification is an oversimplification; macrophage phenotypes are dynamic and context-dependent (112). Critically, their roles may shift across different stages post-MI, implying that interventions targeting macrophages should be tailored to the timing of administration. Translational challenges arise from discrepancies between animal models-such as genetically homogeneous mice with physiological differences from humans and human disease (113). Thus, failed macrophage-targeted therapies may reflect incomplete understanding of their functional plasticity and model limitations, rather than target invalidity. Recent work further reveals an epigenetic layer: RBPJ suppresses H3K9me3 at Stard13/Arsg promoters, enhancing efferocytosis via actin polymerization (114), representing a novel immunomodulatory node. Beyond efferocytosis, the survival of tissue-resident cardiac macrophages is equally critical.

The role of T cells in MI remains controversial. While study demonstrated that DC-mediated CD8⁺ T cell activation exacerbates inflammation and adverse remodeling-an effect attenuated by DC-targeted CTLA-4 nanovesicles, other studies highlight the reparative functions of T cell subsets such as IL-33-responsive Tregs (115, 116). These opposing effects may reflect differences in immune response timing and model systems, further underscoring the need for stage-specific modulation of T cell activity in therapeutic design.

While animal models, particularly mice, have been instrumental in elucidating fundamental mechanisms of post-MI immunity, the translational relevance of these findings is constrained by several methodological limitations. Most studies rely on young, genetically uniform animals under controlled conditions, which do not fully recapitulate the comorbidities such as diabetes, hypertension or age-related immune senescence prevalent in human MI patients (117, 118). Moreover, experimental designs often do not systematically account for the temporal evolution of the immune response, limiting insight into how interventions might perform in the acute vs. chronic phase of MI. For example, the promising role of Treg-specific CD73 in mouse models warrants validation in human cohorts, where immune cell trafficking and metabolic reprogramming may differ (119). Moreover, the overreliance on myeloid-specific or global knockout models may obscure cell-specific mechanisms and compensatory pathways. The emerging technique of scRNA-seq in human MI samples begins to address this by providing unbiased immune cartography, yet functional validation in more humanized systems is urgently needed. Similarly, studies linking splenic extramedullary hematopoiesis to cardiac repair via Ly6C^low^ monocyte production rely on splenectomy or splenic transplantation models (120). These methodologies tend to overlook integrated systemic influences, including bone marrow-derived hematopoiesis and neurohormonal activation, potentially leading to an overestimation of the spleen's autonomous contribution to post-infarct myocardial healing.

The growing field of immunometabolism reveals that metabolic reprogramming, such as the glycolytic shift in inflammatory macrophages, is a key determinant of immune cell function. Beyond glycolysis, *de novo* fatty acid synthesis (FAS) in cardiac macrophages has emerged as a critical driver of post-MI fibrosis. Using spatial metabolomics, Sadaf et al. demonstrated that infarct-resident macrophages upregulate ACLY and FASN, and that myeloid-specific Acly/Fasn deficiency reduces cardiac fibrosis and improves function (121). Mechanistically, macrophage ACLY promotes IL-33 production, which drives expansion of a pathogenic fibroblast subset (Fibroblast 5). This metabolic flexibility exhibits distinct temporal dynamics, indicating that immunometabolic interventions could be optimized for specific time windows after MI. Although significant challenges persist, critical insights from previous discrepancies, together with technological advances, are advancing the field toward directed and temporally precise immune-mediated cardiac repair.

Cellular senescence in the post-MI heart is an emerging yet underdiscussed node in the cardio-immune axis. MI induces senescence in cardiomyocytes, fibroblasts, and endothelial cells, which accumulate and release pro-inflammatory SASP factors, including IL-6, IL-1β, and MMPs, thereby perpetuating chronic inflammation and adverse remodeling (122). Mechanistically, cytosolic DNA from injured cardiomyocytes activates the cGAS-STING pathway in macrophages, driving both senescence and inflammatory polarization (123). Senescent cells also evade immune clearance, exacerbating maladaptive repair. Targeting senescence via senolytics or STING inhibition represents a promising immunomodulatory strategy, though clinical evidence remains preliminary (124).

## 6 Conclusions

This review discusses the pivotal role of immune cells and their spatiotemporal dynamics within both cardiac tissue and immune organs after MI, emphasizing their cooperative regulation of inflammatory and immune responses, and finally proposes treatment strategies for myocardial infarction based on immune regulation. We specifically explore bidirectional crosstalk between the heart and immune system, termed the cardio-immune axis, and its mechanistic contributions to post-infarction pathophysiology. These interorgan communication networks critically modulate the inflammatory milieu and immune reactions triggered by ischemic injury, ultimately determining tissue repair and ventricular remodeling. Collectively, this synthesis elucidates multidimensional systemic pathophysiology in MI by mapping communication networks across organ systems and highlighting their implications for therapeutic targeting.
